# Toward optical coherence tomography on a chip: in vivo three-dimensional human retinal imaging using photonic integrated circuit-based arrayed waveguide gratings

**DOI:** 10.1038/s41377-020-00450-0

**Published:** 2021-01-05

**Authors:** Elisabet A. Rank, Ryan Sentosa, Danielle J. Harper, Matthias Salas, Anna Gaugutz, Dana Seyringer, Stefan Nevlacsil, Alejandro Maese-Novo, Moritz Eggeling, Paul Muellner, Rainer Hainberger, Martin Sagmeister, Jochen Kraft, Rainer A. Leitgeb, Wolfgang Drexler

**Affiliations:** 1 Center for Medical Physics and Biomedical Engineering, Medical University of Vienna, Waehringer Guertel 18-20/4 L, 1090 Vienna, Austria; 2 Research Centre for Microtechnology, Vorarlberg University of Applied Sciences, Hochschulstrasse 1, 6850 Dornbirn, Austria; 3 AIT Austrian Institute of Technology GmbH, Gieffinggasse 4, 1210 Vienna, Austria; 4 ams AG, Tobelbader Strasse 30, 8141 Premstaetten, Austria

**Keywords:** Integrated optics, Biophotonics

## Abstract

In this work, we present a significant step toward in vivo ophthalmic optical coherence tomography and angiography on a photonic integrated chip. The diffraction gratings used in spectral-domain optical coherence tomography can be replaced by photonic integrated circuits comprising an arrayed waveguide grating. Two arrayed waveguide grating designs with 256 channels were tested, which enabled the first chip-based optical coherence tomography and angiography in vivo three-dimensional human retinal measurements. Design 1 supports a bandwidth of 22 nm, with which a sensitivity of up to 91 dB (830 µW*)* and an axial resolution of 10.7 µm was measured. Design 2 supports a bandwidth of 48 nm, with which a sensitivity of 90 dB (480 µW*)* and an axial resolution of 6.5 µm was measured. The silicon nitride-based integrated optical waveguides were fabricated with a fully CMOS-compatible process, which allows their monolithic co-integration on top of an optoelectronic silicon chip. As a benchmark for chip-based optical coherence tomography, tomograms generated by a commercially available clinical spectral-domain optical coherence tomography system were compared to those acquired with on-chip gratings. The similarities in the tomograms demonstrate the significant clinical potential for further integration of optical coherence tomography on a chip system.

## Optical coherence tomography: chip promise

The goal of an optical coherence tomography (OCT) imaging system that is integrated on a photonic chip has taken a step closer to reality. Elisabet Rank and coworkers from Austria have shown that arrayed waveguide gratings (AWGs), integrated-optical devices commonly used to separate different wavelength channels in an optical communications system, can be used to replace diffraction gratings in an OCT system. Several designs of silicon nitride AWGs with 256 channels in the near-infrared were fabricated and then tested in an OCT system which was able to capture in-vivo tomograms and angiography of the human eye’s retina, with comparable quality to a conventional system. The next stage of the OCT-on-a-chip research will focus on exploring the use of multimode interference structures, integrated photodiodes, and compact light sources.

## Introduction

Optical coherence tomography (OCT), the most successful ophthalmological imaging technique to noninvasively visualize the subsurface layers of the retina, has massively advanced in terms of resolution as well as contrast in the past few decades^1^. Today, OCT is considered a standard imaging technique for ophthalmologic care with high scientific, clinical, and economic impact^2^. The commercial standard, spectral-domain OCT (SD-OCT), uses broad bandwidth light that is fed to an interferometer. Light back-reflected from the sample and reference arms interferes, chromatically diverges after passing through a diffraction grating, and is then projected onto a camera. Fourier transformation of the acquired spectrum results in a depth profile of the sample^3^. In recent years, the performance of SD-OCT systems has increased considerably; wider bandwidth light sources improved the axial resolution, while faster cameras enabled shorter acquisition times and therefore opened up the possibility of volumetric imaging^4^.

However, comparably little effort has been made to reduce the size and cost of OCT systems. With a volume of approximately 1 m^3^ and a cost of up to ∼100,000 dollars, an OCT system is, both space and cost-wise, a large investment. Since these parameters are becoming increasingly critical in medical facilities, there is a strong need to lower the costs and miniaturize OCT systems^5^.

To reduce the sizes and costs of OCT systems, one approach is to use smaller and cheaper system components. The usage of off-the-shelf small size optics^6^, low-cost components such as MEMS mirrors for scanning^7,8^, and low-cost 3D printed handheld probe housings^7,9–11^ were reported to be successful measures to reduce the sizes and costs of OCT systems.

Another approach, which still requires more intensive basic and engineering research to develop functional structures for diagnostic OCT applications, is the use of photonic integrated circuits (PICs), i.e., those fabricated using plasma-enhanced chemical vapor deposition. Their compatibility with CMOS fabrication processes is an attractive advantage for OCT application^12^ because this allows for cost-effective and reliable mass fabrication. With their small footprints and monolithic co-integration of several optical and optoelectronic functional building blocks, PICs can substantially reduce the costs and sizes of OCT systems while simultaneously increasing their stability.

With its origin in the telecom regime, PIC development for OCT application started in the range of 1300–1500 nm^13^. Michelson interferometers for 1500 nm^14,15^, Mach–Zehnder interferometers for 1300^16^ and 1550 nm^17^ as well as multimode interferometers for 1300 nm^18^ and polarization splitters for 1550 nm^19^ have been developed for swept-source OCT. Huang et al.^20^ presented an integrated three-layer cascade of 1 × 2 splitters resulting in eight sample arm channels for parallelized sample scanning. Upon implementation in OCT setups, these works reported up to a 91 dB sensitivity (26 mW on the sample^20^) and a 13 µm axial resolution^16^. Further integration of OCT components was shown by Schneider et al.^18^, who additionally integrated photodiodes, achieving a 64 dB sensitivity; Eggleston et al.^21^ presented an integrated interferometer, integrated balanced photodiodes, and a co-packaged MEMS mirror and measured sensitivity of 90 dB for skin imaging. Sancho-Dura et al.^22^ developed a handheld, tablet-like packaged, battery-driven OCT system for skin imaging, including an epiluminescence microscope and a clinical image camera with a total weight of 3 kg. Using frequency multiplexed time-domain OCT, they achieved a system sensitivity of 93 dB (3.5 mW on the sample) and 11 µm axial as well as lateral resolution.

**Fig. 1:**
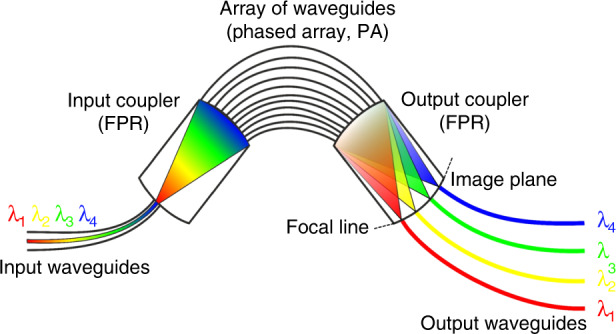
Principle structure of an arrayed waveguide grating. Broad bandwidth light diverges laterally in the input star coupler toward the array of waveguides. There, each waveguide forwards a portion of the input light toward the output star coupler, resulting in different phase delays caused by the different optical path lengths of the individual waveguides. At the focal line on the image plane of the output star coupler, only plane waves with the same phase delay constructively interfere; therefore, each output waveguide forwards individual wavelengths

PIC-based SD-OCT requires more complex photonic building blocks. A key component in conventional SD-OCT is the diffraction grating, which, in combination with focusing optics and a camera, constitutes the spectrometer of the system. In PICs, the discrete diffraction grating can be replaced by an arrayed waveguide grating (AWG)^23^, which is a photonic building block capable of spectral separation of light. Figure 1 shows a schematic drawing of an AWG. It consists of input and output waveguides, input and output star couplers, and an array of waveguides (also called a phased array, PA). AWGs can be used as either a multiplexer or a demultiplexer. In the latter case, broadband light is coupled to the input waveguide, which guides the light towards the input star coupler. The input star coupler effectively acts as a free propagation region, in which the light beam diverges in the lateral direction. The divergent beam coupled to the array of waveguides. The length of the waveguides in the PA linearly increases from one waveguide to the next. Each waveguide in the PA guides a portion of the input light toward the output star coupler, resulting in different phase delays caused by the different optical path lengths of the individual waveguides. At the focal line on the image plane in the output star coupler, only plane waves with the same phase delay constructively interfere. Each output waveguide consequently forwards individual wavelengths, which then can be further redirected towards the end facet of the PIC or toward integrated photodiodes.

Table 1 provides an overview of AWG-based OCT demonstrations reported in the literature. Most reports on AWG-based OCT systems are for the wavelength region of 1300 nm. Nguyen et al.^24^ reported a tomogram of a layered phantom using an integrated AWG with 195 channels at 1300 nm. Akca et al.^25^ further showed a system with an integrated AWG with 125 channels for the 800 nm wavelength region. In 2013, Akca et al.^26^ presented the first in vivo tomogram of human skin (32 times averaged), measuring the sensitivity of 74 dB with 0.5 mW on the sample and a 47 kHz A-scan rate. To the best of our knowledge, there were no publications on AWGs for OCT application for several years until 2019, when Ruis et al.^27^ reported a silicon nitride AWG designed for an on-chip OCT system in the 850 nm wavelength region. Their AWG was fabricated using low-pressure chemical vapor deposition and a cascaded AWG scheme to reduce the size of the AWG while increasing the output channels to 512. A sensitivity of 77 dB was achieved at an A-scan rate of 1 kHz, and the imaging capabilities of the system were demonstrated with a B-scan of four layers of scotch tape (20 times averaged).

**Table 1:** Overview of published work using AWGs for OCT application: sorted by year including wavelength region, waveguide technology, and key features of the systems

Publication date	Wavelength (nm)	Waveguide technology	Features
2011/05^24^	1300	SiON strip	Integrated AWG (195 channels)
			SNR: 75 dB
			19 µm axial resolution
2012/05^25^	800	SiON strip	Integrated AWG for 800 nm (125 channels) and 1300 nm (195 channels)
	1300		25 µm (800 nm) and 20 µm (1300 nm)
2013/07^26^	1300	SiON	Integrated AWG
			SNR: 74 dB at 47 kHz
			7.5 µm axial resolution
2019/01^27^	850	TriPleX Si_3_N_4_	Integrated 50/50 splitter and a cascaded AWG (512 channels)
			Sensitivity: 77 dB at 1 kHz
			6-dB roll-off at 400 µm
			5.9 µm axial resolution

Previously demonstrated implementations of AWG PICs for OCT have the following major drawbacks: They still require laborious packaging because too many external components are still needed. Furthermore, for in vivo imaging, higher sensitivities are necessary to provide a sufficient image acquisition rate without the need for image averaging. Particularly in the case of retinal imaging, image artifacts are often present due to the motion of the eyeball. Minimization of such artifacts can only be performed by increasing the acquisition rate while maintaining sufficient sensitivity at eye-safe light source power levels.

In this work, we present the first in vivo retinal tomograms using AWGs with 256 output channels without the need for time-intensive averaging. The AWGs were fabricated on a fully CMOS-compatible waveguide platform. The CMOS compatibility allows the integration of dedicated photodiodes for each spectral channel. Moreover, the electronics for the entire read-out chain can be on the same chip, rendering an external CCD camera unnecessary. In principle, a light source can be heterogeneously integrated on the chip as well. In addition to the small footprint, these co-integrated components result in one major advantage, particularly for future point-of-care devices, because all major components are on one monolithic semiconductor chip: high mechanical robustness. Moreover, (re)alignment and laborious packaging can be significantly reduced. This directly addresses the aforementioned drawbacks of the important work presented by other groups to date.

The performance of two AWGs, supporting bandwidths of 22 and 48 nm, was evaluated. We provide a comparison of data acquired with each AWG design and with a commercial SD-OCT system that sets a realistic benchmark for the PIC-based OCT systems.

## Results

### AWG and OCT system characterization

Table 2 gives a summary of the measured AWG parameters. Two compact 256-channel AWGs were designed and fabricated.

**Table 2:** Summary of measured AWG and AWG OCT setup parameters used in this study

Parameter	AWG 1	AWG 2
Number of channels	256	256
Wavelength spacing	0.09 nm	0.19 nm
Bandwidth	22 nm	48 nm
Wavelength region	782–804 nm	850–898 nm
Center wavelength	794 nm	875 nm
Mean transmission (33 channels)	−15.51 dB	−11.64 dB
Power on the eye	830 µW	480 µW
A-scan rate	34 kHz/67 kHz	20 kHz
Measured sensitivity	91 dB/88 dB	90 dB
Axial resolution (in soft tissue)	10.7 µm	6.5 µm
Imaging depth	1123 µm	645 µm
6 dB roll-off depth	approx. 625 µm	approx. 380 µm

**Fig. 2:**
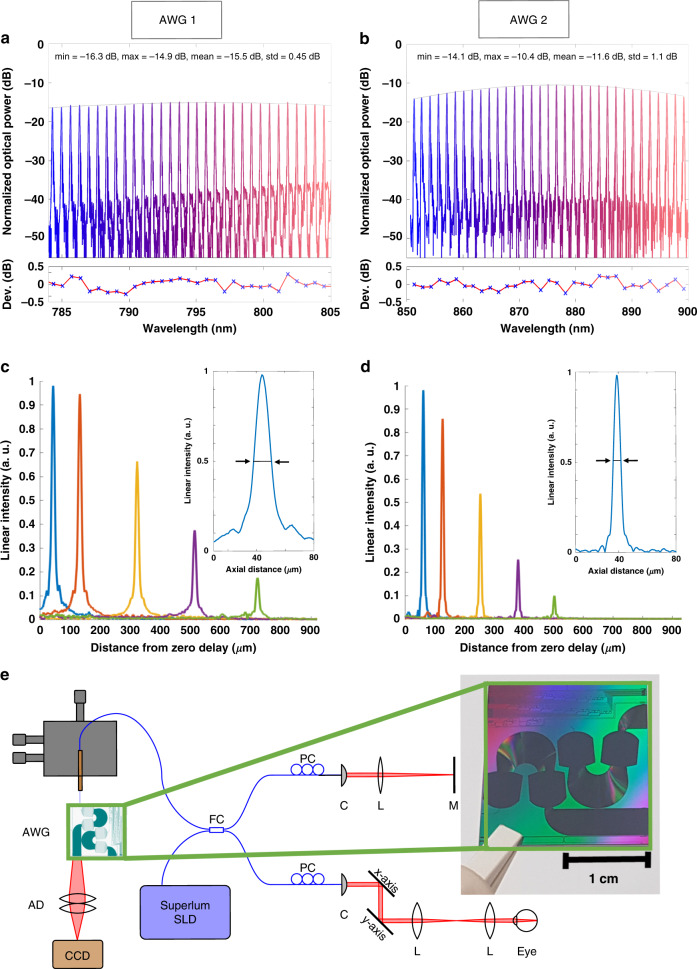
Characterization measurements of the two 256-channel AWGs. Measured spectral characteristics of **a** AWG 1 and **b** AWG 2 for every eighth channel: the minimum, maximum, mean, and standard deviation of the peak powers are provided in the two figures. The thin black line is a polynomial second-order fit to the peaks. This fit shows the AWGs typical spectral envelope, which is different for the two designs. The deviation of the individual peaks from this envelope fit (peak power minus power of the envelope at the peak wavelength) is shown in the two figures below (red lines with blue crosses). The deviation of ~±0.5dB can be explained by the inaccuracy of the fiber alignment with respect to the chip. For the OCT measurements, where no fiber at the output was used, these variations are not present. Sensitivity roll-off measurements of **c** AWG 1 and **d** AWG 2 with the respective axial resolution measurements as insets: 14.5µm in air and 10.7µm in soft tissue (AWG 1) and 8.8µm in air and 6.5µm in soft tissue (AWG 2). **e** Scheme of the SD-OCT on-chip setup: a Superlum SLD fed broadband light to a fiber coupler, and 830μW (AWG 1, a booster amplifier and a 90/10 coupler were used) and 480μW (AWG 2, no booster amplifier and a 50/50 coupler were used) light on the eye interfered with the reference light and was coupled into the on-chip AWG. Projection optics were used to project the light from the PIC end facet onto a CCD camera. FC fiber coupler, PC polarization controller, L lens, C collimator, M mirror, AWG arrayed waveguide grating, AD achromatic doublet

AWG 1 had a center wavelength of 794 nm and a wavelength spacing per output channel of 0.09 nm, resulting in a 22 nm bandwidth. AWG 2 had a center wavelength of 875 nm and a wavelength spacing per output channel of 0.19 nm, resulting in a 48 nm bandwidth. Each AWG measured 13 × 14 mm^2^, and they were realized on a semiconductor chip with a size of 20 × 20 mm^2^, as shown in Fig. 2e. Both AWGs were designed for a center wavelength of 850 nm. However, due to deviations in the fabricated structures and actual refractive indices from the design values, the two AWGs had a shifted central wavelength, which is discussed in more detail in the supplementary information. Therefore, the systems for the two AWGs were optimized individually, resulting in different coupler splitting ratios, powers on the eye, and imaging speeds. These differences need to be considered when interpreting the data acquired with the two systems in terms of the dynamic range. The system differences, however, do not influence the AWG-design-specific parameters such as the axial resolution or signal roll-off with depth. The details of the system differences are described in the “Materials and methods” section.

For optical characterization of the AWG transmission characteristics, the light of a tunable Ti:sapphire laser source (800–900 nm) was coupled to an inverted taper with a tip size of 160 × 160 nm^2^ using a polarization-maintaining fiber. The coupling loss was ~2.5 dB per coupling event. The term coupling event describes solely the coupling from the fiber to the waveguide or vice versa. All losses caused by this single coupling event, including all losses caused by the coupling structure on the chip, are considered. All losses not directly caused by the coupling event, such as propagation losses in the attached waveguides, are excluded. The propagation loss for the TM mode amounted to ~0.5 dB/cm. To characterize the transmission losses, light from the individual AWG output channels was collected with a standard single-mode fiber. The transmitted power was measured with an optical power meter for channels 1 and 8 and then every eighth output channel thereafter (i.e., channels 1, 8, 16, 24, …, 256), which resulted in 33 measurement points over the whole spectrum. The alignment was optimized with a piezo-driven auto-alignment system to achieve optimal coupling. The obtained transmission loss was normalized to the wavelength-dependent optical power of the laser source. Figure 2 shows the measured characteristics of AWG 1 (Fig. 2a) and AWG 2 (Fig. 2b). The mean transmission over the 33 measurements was calculated to be −15.51 dB (AWG 1) and −11.64 dB (AWG 2). While this setup had two fiber—PIC coupling events (one on the input side and one on the output side), the OCT setup included coupling only once from the fiber to the PIC (on the input side of the AWG). On the output side of the PIC, the light was projected via a pair of achromatic lenses. These differences reduced the transmission losses by 1–2 dB for the OCT setup compared to the above-described characterization setup.

The AWGs were combined with a fiber-based interferometer as described in the “Materials and methods” section. The maximum sensitivity and roll-off with the depth of the systems were measured. A neutral density filter (NDC-50C-2M-B, Thorlabs Inc., USA) with a measured attenuation of 17.3 dB for AWG 1 and 15.4 dB for AWG 2 was placed in front of a focusing lens and a mirror in the sample arm. The maximum signal-to-noise ratio (SNR) of the point spread function (PSF) that could be achieved was calculated, as proposed in ref. ^29^, to be 53.7 dB (AWG 1, 67 kHz), 56.2 dB (AWG 1, 34 kHz), and 59.5 dB (AWG 2, 20 kHz). For AWG 1, a maximum sensitivity of 91 dB with 830 µW on the sample and a 34 kHz A-scan rate was calculated by adding double the attenuation factor (=34.7 dB, to account for double path attenuation) introduced by the neutral density filter to the measured SNR. The same procedure resulted in insensitivities of 88 dB with 830 µW on the sample and a 67 kHz A-scan rate (AWG 1) and 90 dB with 480 µW on the sample and a 20 kHz A-scan rate (AWG 2).

Figure 2c, d shows the sensitivity roll-off with depth for both systems. The 6-dB roll-off depth was measured to be approximately 625 µm for AWG 1, with an overall imaging depth of 1123 µm, and approximately 380 µm for AWG 2, with an overall imaging depth of 645 µm. The axial resolution of the system was measured by calculating the FWHM of the PSF, as indicated in the insets of Fig. 2c for AWG 1 and Fig. 2d for AWG 2. The FWHM for each peak of the five measurement points in depth was calculated. A mean axial resolution of (14.5 ± 0.36) µm in the air (theoretically calculated 12.7 µm) for AWG 1 and (8.8 ± 0.35) µm in the air (theoretically calculated 7.0 µm) for AWG 2 was measured, which correspond to 10.7 µm and 6.5 µm in scattering tissue, respectively, assuming a refractive index of 1.3549^30^.

### In vivo retinal imaging

To identify the capabilities of the system in imaging living tissue, the right eye of a healthy volunteer was investigated with both AWG systems. Imaging was performed under a protocol approved by the institutional ethics committee of the Medical University of Vienna and followed the tenets of the Declaration of Helsinki (EK nr: 253/2004). Informed consent was obtained after explaining the form and nature of the measurements. With eye-safe light source powers of 830 µW (AWG 1) and 480 µW (AWG 2) on the cornea, the retina of the volunteer was scanned with acquisition rates of 34 kHz and 67 kHz (AWG 1) and 20 kHz (AWG 2).

**Fig. 3:**
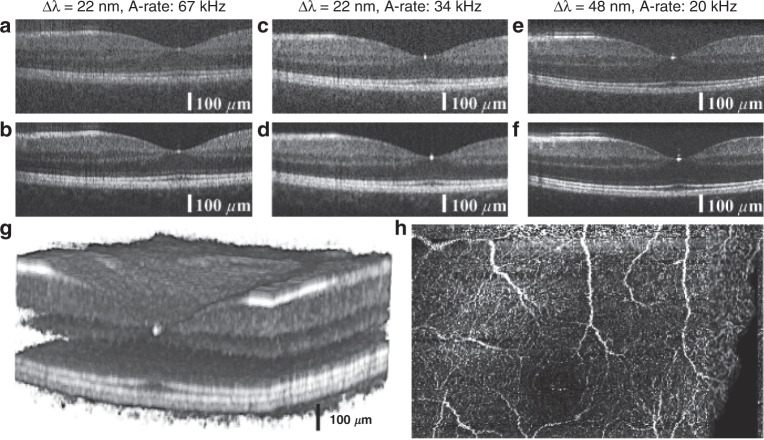
B-scans of a healthy retina in the foveal region. **a** Unaveraged and **b** five times averaged fovea acquired with AWG 1 at 67kHz. **c** Unaveraged and **d** five times averaged fovea acquired with AWG 1 at 34kHz. **e** Unaveraged and **f** five times averaged fovea acquired with AWG 2 at 20kHz. In areas perpendicular to the scanning beam, strong reflection induces visible side lobes. **g** 3D representation of the retina in the foveal region acquired with AWG 1 at 67kHz, and **h** corresponding OCTA image calculated from the volume using five B-scan repetitions. The black area on the right side of the angiogram corresponds to missing data due to motion correction in the lateral direction

Figure 3 shows an overview of the three different imaging scenarios: Fig. 3a, b shows the fovea as unaveraged and five times averaged images, respectively. These images were acquired with AWG 1 at 67 kHz, the highest speed possible with the camera. Figure 3c, d shows the same area acquired with AWG 1 at 34 kHz, which increases the dynamic range of the tomograms. Figure 3e, f shows the same fovea imaged with AWG 2 at a 20 kHz A-scan rate with 480 µW incidents on the eye, where a difference in bandwidth and axial resolution can be noted. Additionally, a steeper signal roll-off can be observed in Fig. 3e, f, where the choroid layer is almost not visible compared with the tomograms of AWG 1. By acquiring a volume through several B-scans at the same position but with differences in time, an OCT angiography dataset can be obtained. Figure 3g shows the 3D volume of the region around the fovea acquired with AWG 1 at a 67 kHz A-scan rate. The volume was acquired with five repetitions per B-scan, from which the corresponding OCT angiogram was calculated. Maximum intensity projection was performed over the depth of each A-scan to visualize the vasculature in the foveal region, as shown in Fig. 3h.

As the human retina in the foveal region does not usually exceed a thickness of 300 µm^31^, the retinal image of the subject could be aligned to be within the 6-dB roll-off region to achieve the best possible contrast. However, it is not only the macular region that is of clinical relevance. The optic nerve excavation towards the brain well exceeds the thickness of the retina itself, and therefore, deeper imaging is required to visualize it. Cup-to-disc ratios of the optic nerve head are used to monitor glaucoma patients^32^, which requires the full optic nerve cup to be visible in the tomograms. Good signal quality at deeper depths is also required for patient imaging. If the patient cannot fixate very well, then higher axial movements are expected, which results in imaging further away from the zero delays. To investigate the impact of the strong signal roll-off in the AWG systems on the contrast of tomograms, the subject’s optic nerve cup was imaged.

**Fig. 4:**
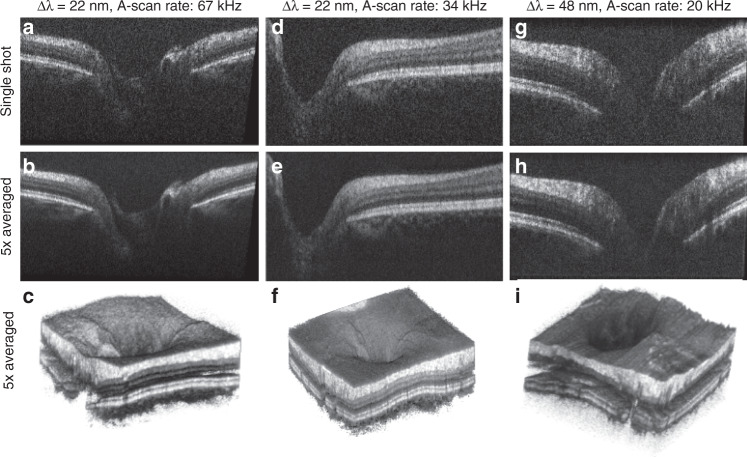
In vivo measurements of a healthy retina: in the region of the optic nerve head imaged with **a**–**c** AWG 1 at 67kHz, **d**–**f** AWG 1 at 34kHz, and **g**–**i** AWG 2 at 20kHz. All data are an average of three registered B-scans

Figure 4 shows a summary of the acquired volumes and selected B-scans in the area of the optic nerve head. Each tomogram is an average of three registered B-scans; the 200 B-scans in the 3D volumes are also an average of three B-scans each. A B-scan consists of 400 A-scans. Figure 4c, f, i shows 3D volumes of the optic nerve depression obtained with AWG 1 at 67 kHz, AWG 1 at 34 kHz, and AWG 2 at 20 kHz, respectively. From these volumes, we selected two types of B-scans: Fig. 4a, d, g displays to nerve head to determine whether the optic nerve cup can be visualized. AWG 1, having a slightly better roll-off than AWG 2, shows the entire cup in Fig. 4a, d, which could be used for glaucoma monitoring. AWG 2, as shown in Fig. 4g, fails to visualize the cup fully due to the high signal roll-off, though the thickened retinal nerve fiber layer shown in Fig. 4h is resolved with good contrast using AWG 2. Tomograms in this area acquired with AWG 1, however, still show better signal with depth (Fig. 4b, e), as the choroid layer is visible, whereas it is almost not visible when imaged with AWG 2 (Fig. 4h).

**Fig. 5:**
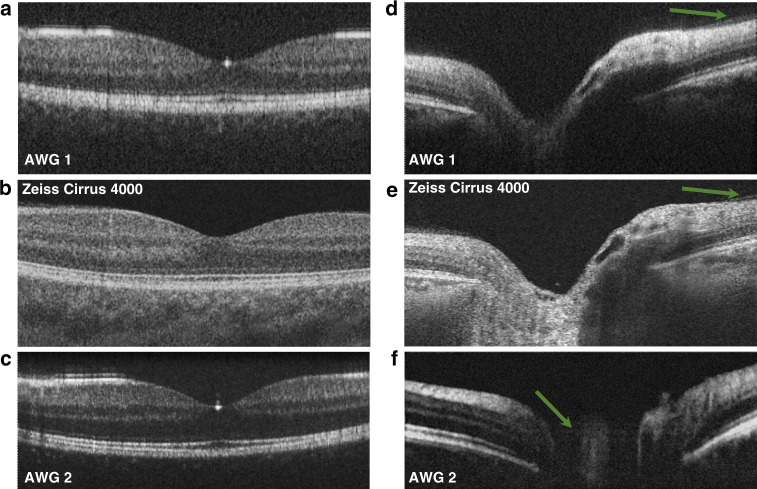
OCT on a PIC system in comparison to a commercial OCT device. Direct comparison of the tomograms acquired with the SD-OCT on a PIC system with tomograms of the same eye acquired with a Zeiss Cirrus 4000. **a**, **d** Acquired with AWG 1 at 34kHz; **b**, **e** acquired with the Zeiss Cirrus 4000; **c**, **f** acquired with AWG 2 at 20kHz. The reduced imaging depth with AWG 2 can especially be observed, as the optic disc cup has poor contrast and an aliasing effect occurs (as indicated with the green arrow) in (**f**). The green arrows in **d** and **e** indicate the boundary of the vitreous

Finally, a commercial system was used as a benchmark for the AWG systems. The same eye was imaged with an SD-OCT Cirrus 4000 (Zeiss, Jena, Germany), which acquires data with an A-scan rate of 27 kHz and a 5 µm axial resolution. A five-line raster was chosen to image the fovea as well as the area at the optic nerve head. From each area, one of the five acquired tomograms was selected and saved as a greyscale image. Figure 5 shows a comparison of the tomograms acquired with the AWG systems with those acquired with the commercial OCT system (Zeiss Cirrus 4000): The larger field of view of the Cirrus tomograms was cropped in Fig. 5b, e to match the field of view of the tomograms acquired with the AWG setups. The individual layers of the retina are as distinguishable as in the commercial tomograms in both cases. Even the external limiting membrane in the fovea tomogram in Fig. 5a, obtained with the reduced bandwidth of AWG 1, is well distinguishable, although the contrast is slightly reduced. In addition, as shown in Fig. 5c obtained with AWG 2, the external limiting membrane is still visible, albeit with less contrast than in the commercial tomograms. Comparing the commercially acquired tomogram of the optic nerve cup in Fig. 5e with that acquired with the AWG 1 setup in Fig. 5d, it can be noted that the lines indicated by the green arrow are the boundary of the vitreous, with slightly less resolution and therefore less contrast in the AWG system. The steep sensitivity roll-off of the AWGs, however, can be noted by the reduced contrast of the choroid. An even stronger difference in signal loss with depth can be observed when comparing the commercial tomograms with the AWG 2 tomograms in Fig. 5c, f. While the retinal layers can be distinguished well in the foveal region in Fig. 5c, the steep roll-off is apparent for the optic nerve cup in Fig. 5f. An aliasing effect due to the limited overall imaging depth occurs, as indicated by the green arrow in Fig. 5f.

## Discussion

In this work, we showed for the first time PIC-based in vivo retinal OCT imaging. Axial resolutions of 10.7 µm (AWG 1) and 6.5 µm (AWG 2) were measured, which enable the systems to resolve the individual layers of the retina. The comparison with a commercial SD-OCT system provided a realistic benchmark for the acquired tomograms. Non-averaged imaging is possible due to the sufficient system sensitivities of both systems. OCTA, a clinically important add-on for OCT^33^, was also demonstrated in this work. Our data were fed to an OCTA algorithm that had been optimized for standard OCT data, with a sensitivity of over 95 dB, acquired at a B-scan rate superior to 400 Hz with a screening of at least a 30° field of view. For the OCT engine described in this work, it was necessary to acquire at least five repetitions to retrieve angiographic contrast. The total acquisition time of the volume (several seconds) comes with unwanted distortions in the en face projection introduced by eye motion. This effect is magnified by the small field of view where a small movement (>20 µm) could blur out small capillaries. Therefore, to retrieve the original morphology of the vasculature and in some areas visualize small capillaries, a motion correction algorithm based on the cross-correlation of consecutive slow-axis position B-scans was added to the OCTA algorithm pipeline. Further improvement can be achieved by implementing a real-time tracking device in combination with motion correction technology, as proposed in ref. ^34^.

Compared to conventional diffraction gratings, our AWGs have a lower number of channels, smaller bandwidths, and higher transmission losses. However, the presented results show for the first time that AWG-based OCT for in vivo retinal imaging is capable of generating tomograms with clinically acceptable contrast and resolution without the need for high numbers of averaging. This is an important step towards the integration and therefore miniaturization of OCT devices. By reducing the imaging speed by a factor of two in the system with AWG 1, it can be seen in Fig. 4a, d that the overall signal increase especially helps with the signal contrast at depth. However, for OCTA calculation, eye motion is greater at slower imaging speeds, and it might be too high to resolve vasculature. Therefore, depending on the intended use, systems with such sensitivities need to be accordingly adapted in terms of imaging speed.

With over 10 dB higher sensitivities than previous attempts found in the literature, it is of high interest to discuss the parameters that might be responsible for such large differences. Due to a lack of information about the AWGs and their transmission losses in previously published work, it is difficult to determine what sets our AWGs apart from others in achieving sensitivities appropriate for in vivo imaging. However, during the development of an AWG-based OCT system, we found the proper projection from the AWG output to the CCD to be a crucial parameter: transmission losses of over 10 dB per AWG significantly raise the importance of optimum projection of the waveguide output onto the CCD. To minimize the complexity of the projection optics, the separation of the output waveguides was set to 14 µm, which equals the pixel pitch of the used CCD. It was therefore possible to use a rather simple 1:1 projection from the AWG to the CCD. Careful alignment of the CCD within five degrees of freedom (*x*, *y*, *z*, tip, tilt) was also found to have a significant influence on the system performance. During the CCD alignment, it is important not only to align towards the maximum signal strength on the camera but also to ensure that the correct number of pixels, in this case, 256, are illuminated. Finally, the maximum modulation depth of the spectral interference pattern measured with a mirror as a sample placed close to the zero delays was a useful indicator for proper projection during adjustment of the abovementioned five degrees of freedom.

As our booster amplifier does not amplify wavelengths over ~870 nm (as shown in more detail in the materials and methods section), we could not supply the maximum eye-safe light power to the cornea in the AWG 2 setup within this study. Even with the usage of a 50/50 coupler, the power on the cornea was well below this value, which influences the overall system sensitivity. It must be emphasized that this is not a drawback of the AWG itself. In fact, AWG 2 was measured to have lower transmission losses than AWG 1 and therefore would transmit more signal to the camera. Once the above-described optimization processes are carried out, uncertainties in the wavelength regions can be minimized, and system components such as the light source and booster amplifier can be appropriately chosen to realize optimum system design (e.g., a 90/10 coupler and the maximum eye-safe light power on the cornea). While the transmission losses, system (coupler) design, and power on the eye are sensitivity-related parameters, these differences do not affect the comparability of the two AWG designs: Channel spacings of 0.09 nm (AWG 1) and 0.19 nm (AWG 2) were realized in the two 256-channel AWGs, which resulted in different bandwidths and hence axial resolutions as well as signal roll-off with depth. These parameters are not related to the system sensitivity, therefore are not changed in different setup designs (assuming optimal coupling of light from the AWG to the CCD) and can be compared directly.

**Fig. 6:**
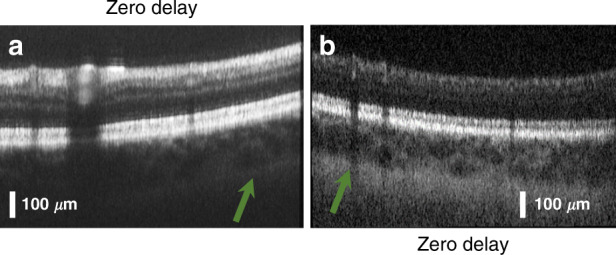
Signal roll-off with depth compensation for AWG 2. **a** Average of 100 registered B-scans; **b** average of three registered B-scans, where the retina was aligned so that the sclera was close to the zero delays. The green arrows in the tomograms indicate the choroid/sclera junction

The two AWGs show either good, i.e., low, signal roll-off (AWG 1) or good axial resolution (AWG 2) for ophthalmic imaging. While an axial resolution of 10.7 µm still resolves all the individual layers of the retina and therefore could be useful in clinical diagnosis, the steep signal roll-off of AWG 2 limits clinical application to the investigation of the upper layers of the retina, as the optic nerve cup has poor contrast at depth (Fig. 4c vs. Fig. 4d). If deeper layers of the retina are of interest, then there are still options to visualize these using AWG 2: Averaging is an effective technique of increasing the contrasts within tomograms. Figure 6a shows an average of 100 B-scans at the same location, and the arrow indicates that the choroid/sclera junction appears with higher contrast. However, averaging is a computational and time-intensive technique to enhance weak signals. Enhanced depth imaging, as introduced by Spaide et al.,^35^ maybe a preferable approach: The subject’s retina is aligned with the choroid/sclera junction close to the zero delays, where the system achieves higher sensitivities. Figure 6b shows an average of three registered B-scans obtained using this approach. The choroid/sclera junction exhibits good contrast, even with low numbers of averaging. Even so, the upper layers of the retina show poorer contrast, as these are now located at depths of low sensitivity. The signal loss with depth can therefore only be compensated by increasing the measurement time or sacrificing the contrast in other layers.

AWG 2 may therefore be a better choice for microscopic OCT on PIC systems in which steady samples (such as excised tissue samples) can be aligned to be within the depth of good signal intensity. Such studies often do not require high imaging depths (e.g., zebrafish and investigation of epithelial layers) but require rather fine axial resolution.

Nonetheless, the increased axial resolution of AWG 2 (6.5 µm) is of immediate clinical interest, as finer resolution supports early-stage disease diagnosis such as retinal detachment or drusen formation in age-related macular degeneration.^36^

In the current designs, the imaging performance of the two AWGs shows a trade-off between imaging depth and axial resolution, i.e., AWG 1 has superior imaging depth, whereas AWG 2 has a finer axial resolution. Further design optimization desirable for ophthalmic OCT would result in an increased clinical significance of AWGs for OCT. Increasing the number of output channels to 512 while retaining the wavelength spacing similar to that of AWG 1 (~0.09 nm) would result in an AWG-based OCT system with an imaging depth comparable to that of AWG 1 and an axial resolution comparable to that of AWG 2. Such an AWG would therefore be a further step toward AWG-based OCT systems with performance close to that of commercial OCT systems.

The AWGs show a free spectral range of ~40 nm within the wavelength region of 800–900 nm. This means that identical spectra occur repeatedly with a spacing of 40 nm (see Supplementary information). For AWG 1, one peak of the central channel is at a wavelength of ~881 nm, whereas for AWG 2, one peak is at ~875 nm. The reason for the shift of 6 nm between the two types is the different impacts of the fabrication variations on the two different AWG designs (see Supplementary information for details). Moreover, the peak of the center channel exists at ~834 nm, which has a shift of 16 nm compared to the design wavelength of 850 nm. The reason for this shift is the mismatch between the design parameters and the fabricated structures (e.g., waveguide width, waveguide thickness). As described in the supplementary information section in more detail, this is presumably a constant offset. By analyzing the AWG characterization results, and adapted AWG design can be elaborated to compensate for this shift to a large extent in subsequent fabrication runs. Corresponding simulations were performed, revealing that the necessary shift of 16 nm can be achieved by reducing the length of the input and output couplers (see Fig. 1) by 22.38 µm. In addition, the path length difference of the waveguides in the PA must be reduced by 150 nm. The change in the two parameters can be well controlled by current fabrication technology, while the impact on the overall layout of the AWG is negligible. This means that no unintended change of other AWG characteristics is expected. However, it must be mentioned that for the commercialization of these PICs, even a shift of several nanometers is acceptable as long as the spectrum of the AWG is still fully covered by the final light source. If all other AWG characteristics remain the same (number of channels, channel spacing, losses, crosstalk), then such a shift of the entire spectrum will have no significant impact on the OCT performance, which is discussed further in more detail in the Supplementary Information.

**Fig. 7:**
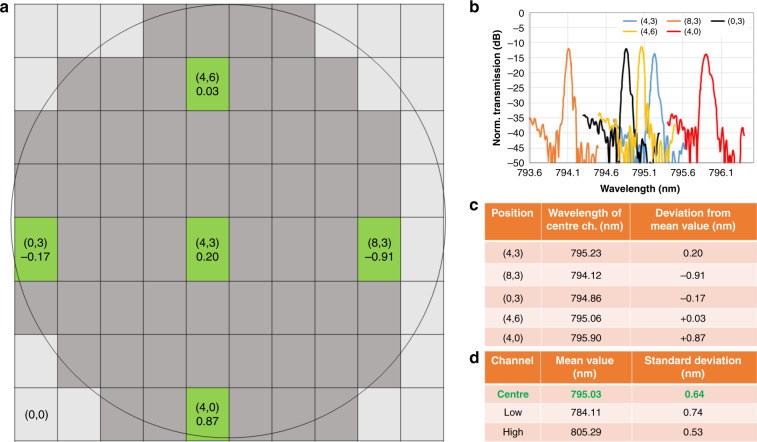
Intra-wafer variation of the AWG 1 characteristics. **a** A schematic of the wafer. Five samples at five positions (highlighted in green) were measured. The numbers in the brackets are the *x* and *y* coordinates on the wafer (*x*,*y*) starting with (0,0). The number below is the difference between the central channel wavelength and the mean wavelength of the five measured AWGs across the wafer. Dark gray boxes indicate useful AWGs on the circular wafer. In **b**, all five center channels are plotted. **c** Summarizes the central wavelength for the individual AWGs as well as the deviation from the mean wavelength of the five center channels. **d** Summarizes the mean value and standard deviation of the center, lowest and highest channels across the five AWGs

Preselection of the chips emerged from the determination of the intra-wafer variations, which was performed prior to the OCT measurements. To minimize the effort for OCT measurements, only the best chips were used. Nevertheless, intra-wafer variations were investigated as summarized in Fig. 7: AWGs were measured at five different wafer positions; see Fig. 7a. In Fig. 7b, the five central channels are plotted, showing a variation of ~1 nm across the wafer. Figure 7c summarizes the central channel wavelength as well as the deviation from the mean wavelength across the wafer, and Fig. 7d summarizes the mean wavelengths for the center, lowest and highest channels, and their standard deviation. As noted in more detail in the supplementary information, a wavelength variation of ~1 nm will not prevent future commercialization. The possible cause of the variations and a strategy to further reduce them are also summarized in the Supplementary section.

This study strongly focused on the characterization of AWGs for OCT application to determine whether AWGs in principle perform well enough for in vivo imaging. For further integration of an OCT system, multimode interference (MMI) structures acting as interference units were tested and optimized separately and will be implemented in the next step. For these MMIs, we demonstrated insertion losses below 0.6 dB, an imbalance of the two outputs below 0.4 dB, and a phase error less than 4°. All values hold over the wavelength range of 800–900 nm. Furthermore, integrated photodiodes^37^ were designed and tested, which will also be implemented in a further step. For further stability of the system, a gluing process to fix fibers on the PIC edge was established as described in ref. ^12^. For low-cost and small-scale scanning, MEMS scanners are a promising option that has also already been used successfully in other small-footprint and low-cost OCT systems^7,8,10,11^. The usage of a compact light source with a footprint of ~10 × 10 cm including a 14-pin butterfly combi-SLED module on an OEM driver board, as introduced in ref. ^38^, will further reduce the overall size of future AWG-based OCT systems.

## Materials and methods

### Arrayed waveguide grating

The PIC was fabricated on a 200 mm standard silicon wafer, as used in the semiconductor industry. The waveguide layer stack consisted of silica acting as a lower cladding with a thickness of 5 µm deposited by means of a chemical vapor deposition process with a temperature below 400 °C. Next, the waveguide core layer (silicon nitride) with a thickness of 160 nm was deposited by means of plasma-enhanced chemical vapor deposition (<400 °C). This was followed by a deep UV photolithography process employing an i-line stepper. Approximately fifty copies of the e-beam written mask of the waveguide structures were realized on a single wafer. For patterning, a chemical dry etches step was carried out. The etching process completely removes the remaining silicon nitride, leaving only the waveguide structures with rectangular cross-sections. With this process, a feature size of ~160 nm was achieved. The thickness of the waveguides was measured in-line with a scanning electron microscope after both mask development and etching. The resulting wire waveguides had a cross-section of 800 × 160 nm^2^. At the edge of the chip, a tip with a cross-section of 160 × 160 nm^2^ enabled efficient coupling to/from the fiber. Next, another silica layer of 4 µm thickness was deposited on top of the waveguides, again by means of a chemical vapor deposition step (<400 °C). Finally, the individual chips were separated with precision sawing. For this, an etched trench avoided chunking at the waveguide edge.

The two 256-channel AWGs were designed to have a 0.1 nm wavelength spacing (AWG 1) or a 0.2 nm wavelength spacing (AWG 2) at a center wavelength of 850 nm. The design parameters, indicated in Fig. 1, were calculated using the tool described in ref. ^39^ and were based on previous studies of AWG designs^40,41^. The AWGs were fabricated at AMS AG (Premstaetten, Austria) using standard CMOS processes, including plasma-enhanced chemical vapor deposition. Unlike low-pressure chemical vapor deposition, which is not CMOS compatible due to the high temperatures involved, with plasma-enhanced chemical vapor deposition, the photonic building blocks are CMOS compatible and can therefore be monolithically co-integrated on one chip with photodiodes and read-out electronics, resulting in a device of only 20 × 20 mm^2^ in size. A description of what such a CMOS-compatible photonic process flow could look like can be found in ref. ^42^. The inset of Fig. 2e shows a photograph of two AWGs on one PIC with a structure size of 13 × 14 mm^2^ per AWG. The other structures above the AWGs are test waveguides for the characterization of the wafer. A detailed description of the AWG design and fabrication process is provided in Seyringer et al.^43^. The AWGs have free spectral ranges of 40 and 45 nm. This means that peaks for a certain channel occur repeatedly with spacings of 40 and 45 nm within the wavelength range of 800–900 nm (for further information, see the Supplementary Information). The two fabricated AWGs were ultimately used at different spectral ranges to have spectral overlap with the used SLD and booster amplifier: AWG 1 at a center wavelength of 794 nm and AWG 2 at 875 nm.

### OCT systems

**Fig. 8:**
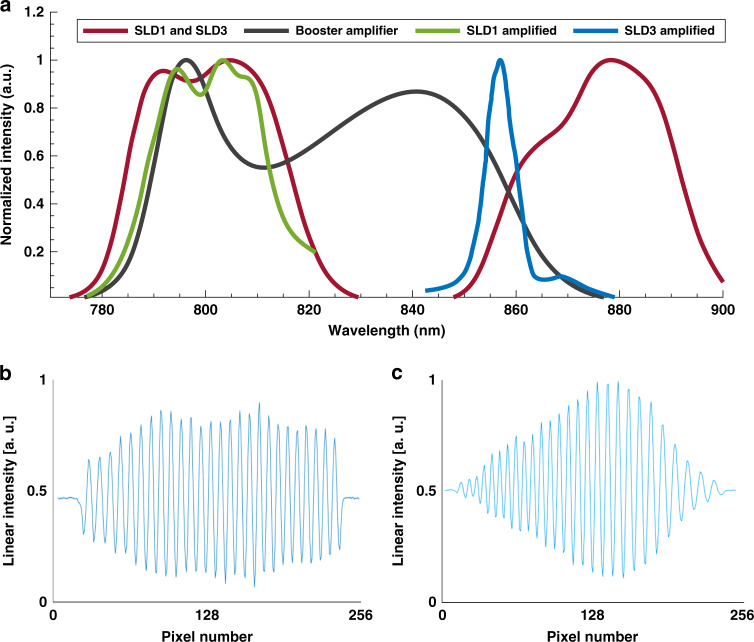
Spectra of the used light sources. **a** Spectra of the Superlum SLD 1 (red, ~780–~830nm), Superlum SLD 3 (red, ~850–~900nm), and booster amplifier (black). The boosted spectrum of SLD 1 is plotted in green; the boosted spectrum of SLD 3 is plotted in blue; due to the insufficient wavelength support, the booster amplifier was not used for the AWG 2 setup, and SLD 3 without the booster amplifier was used instead. **b** Interference pattern of the AWG 1 setup; the envelope represents a rather flat and slightly modulated envelope, as expected from the green spectral shape in (**a**). **c** Interference pattern of the AWG 2 setup: the envelope represents the spectral shape of SLD 3 in (**a**) (red, ~850nm–~900nm)

The setups were based on an 840 nm Superlum SLD (BroadLighter T840, Superlum, Ireland) with three selectable and combinable SLDs. The bandwidth of all three SLDs combined is 120 nm, and the spectrum of the two AWGs could be covered by either SLD 1 (AWG 1) or SLD 3 (AWG 2). Figure 8a shows the spectrum of the Superlum BroadLighter when SLD 1 and SLD 3 are turned on (red line). Furthermore, the spectrum supported by the booster amplifier is plotted in black. The booster amplifier does not cover the full spectrum required for AWG 2. However, SLD 3 was the only SLD supporting the full bandwidth of AWG 2. Finally, the SLDs were boosted by the booster amplifier: the amplified SLD 1 spectrum is plotted in green; the amplified SLD 3 spectrum is plotted in blue. It can be seen that the booster amplifier cuts off the bandwidth of SLD 3 and therefore could not be used for the AWG 2 setup. Each AWG setup was therefore optimized individually to achieve the best possible performance in terms of sensitivity for the individual AWG designs. Figure 8b, c shows the interference patterns of AWG 1 and AWG 2, respectively, at an optical path length difference of ~50 µm, with their envelopes representing the spectral shape of the input light.

### OCT system AWG 1

Figure 2e shows a schematic drawing of the custom-built setup. As AWG 1 supports the wavelength region of 782–804 nm, SLD 1 (~770–~825 nm) could be used and amplified by the booster amplifier to achieve maximum safe power levels on the cornea when using a coupler with a splitting ratio of 90/10 (HI-780 fiber, 840 nm, Gould, Millersville, USA). Ten percent of the power, 830 µW, was incident on the eye in the sample arm, while 90% was sent to the reference arm. The collimated beam (collimator F220APC-850, Thorlabs, USA) with a beam diameter of 2.41 mm in the sample arm was reflected by a set of X–Y galvanometric scanners (621 OH, Cambridge Technology Inc., USA) and then traversed a telescope consisting of two lenses (AC508-100-B and AC508-075-B, Thorlabs Inc., USA), which imaged the pivot point of the scanners onto the pupil of the eye with a beam diameter of 1.9 mm. Polarization paddles (FPC560, Thorlabs Inc., USA) in the sample and reference arms were used to match the two arm polarization states. Light back-reflected from both arms interfered in the fiber coupler and was coupled into the on-PIC AWG to make the bandwidth diverge into individual wavelengths. To couple, the light into the PIC, the FC/APC connector of the fiber carrying the interfered light was removed, and the tube was stripped off. The fiber end was then cleaved at a 0° angle and mounted in a fiber chuck (HFC005, Thorlabs Inc., USA) on an XYZ linear translation stage (M-562-XYZ, Newport Corporation, Irvine, USA) for precise alignment of the fiber with the PIC end facet. An achromatic lens pair (MAP105050-B, Thorlabs Inc., USA) with a magnification ratio of 1:1 projected the individual wavelengths from the end facet of the PIC (output waveguide spacing = 14 µm) onto a CCD camera (e2v AViiVA EM4CL 2014, Essex, UK) with two rows of 2048 pixels, each measuring 14 µm × 14 µm. The camera was mounted on a translational stage with five degrees of freedom (ULTRAlign 561D and ULTRAlign M-561-TILT, Newport Corporation, Irvine, USA) for optimum alignment of the camera in the focal plane of the projected output light. With 256 output channels, AWG 1 forwarded ∼0.09 nm to each pixel.

### OCT system AWG 2

As shown in Fig. 8a, the wavelength region of AWG 2 (850–898 nm) is not fully supported by the booster amplifier, which results in reduced spectral bandwidth when boosting SLD 3 (blue graph). Therefore, the booster amplifier could not be used for this setup. SLD 3 of the light source, which supports the bandwidth of AWG 2 (as shown in Fig. 8a), was fed to a coupler with a splitting ratio of 50/50 (HI-780 fiber, 840 nm, Gould, Millersville, USA) to achieve the maximum possible power on the cornea. Total power of 480 µW was incident on the eye. All other setup components were identical to those listed in the setup description for AWG 1.

However, the different splitting ratio and different power incident on the eye influence the performance of the setups in terms of sensitivity. Furthermore, the insertion losses of AWG 1 and AWG 2 were measured to be different, which also influenced the sensitivity. Table 3 summarizes the sensitivity-related differences between the two systems.

**Table 3:** Summary of sensitivity-related differences in the two AWG setups: Setup AWG 1 uses a 90/10 splitter and a booster amplifier; measurements were taken at A-scan rates of 67 and 34 kHz. Setup AWG 2 uses a 50/50 splitter and no booster amplifier and was operated at a 20 kHz A-scan rate

Parameter	AWG 1 setup	AWG 2 setup	Difference	
AWG transmission	−14.51 dB	−11.64 dB	+2.9 dB	
Power on sample	830 µW	480 µW	−2.4 dB	
Splitting toward AWG	90%	50%	−2.6 dB	
		Compared to	67 kHz	34 kHz
A-scan rate	67 kHz/34 kHz	20 kHz	+5.3 dB	+2.2 dB
Total			+3.2 dB	+0.1 dB
Measured sensitivities	88 dB/91 dB	90 dB		

AWG 2 was measured to have fewer insertion losses, which resulted in a relative sensitivity improvement of +2.9 dB. Less power on the cornea resulted in an expected sensitivity drop of −2.4 dB. While in the setup with AWG 1, 90% of the light reflected from the retina was forwarded towards the detector, in the setup for AWG 2, only 50% was forwarded towards the detector, which accounts for the −2.6 dB sensitivity loss in the AWG 2 setup. Summing up these differences, the setup with AWG 2 was expected to perform 2.1 dB worse than AWG 1, assuming the imaging speed to be the same in both setups. Considering all sensitivity-related system differences between AWG 1 and AWG 2, we limited the AWG 2 setup to an imaging speed of 20 kHz. Compared to the imaging speeds selected for the AWG 1 setup, driving the setup with AWG 2 at 20 kHz gains 5.3 dB (in the case of 67 kHz, AWG 1) and 2.2 dB (in the case of 34 kHz, AWG 1) insensitivity. Therefore, the AWG 2 setup at a 20k Hz A-scan rate was expected to have a 3.2 dB higher sensitivity than AWG 1 at 67 kHz and a 0.1 dB higher sensitivity than AWG 1 at 34 kHz, i.e., the sensitivity in the range of ~90 dB (required for in vivo imaging) was expected. For AWG 1, the maximum possible imaging speed of 67 kHz (limited by the read-out speed of the camera of 70 kHz and additional fly-back time needed by the galvanometric mirrors) was needed for OCTA calculation. For a higher dynamic range, we chose to also drive the OCT setup with AWG 1 at half the imaging speed.

### Data acquisition and post-processing

A field of view of 15 × 15° with a sampling of 400 × 200 pixels was acquired for each measurement. For volume and OCTA data acquisition, three and five B-scans per position were acquired for averaging purposes and OCTA calculation, respectively.

Data from the camera were sent to the computer via a frame grabber (PCIe 1430, National Instruments, USA). The acquisition was synchronized to the galvanometric scanners using a connector box (BNC-2120, National Instruments, USA) and controlled by MATLAB (Version R2015b, 8.6.0.267246, Mathworks Inc., USA). The integration time of the camera was set to 50, 30, or 15 µs, which resulted in A-scan rates of 20 kHz (AWG 2), 34 kHz (AWG 1), and 67 kHz (AWG 1), respectively.

In the first post-processing step, the background of the acquired data was removed by subtracting the median spectrum of the entire B-scan. To reduce side lobes introduced by the non-Gaussian shape of the spectra, the data were first normalized by dividing them by the average of the background spectrum. The spectral data were then resampled to be linear in k-space following the resampling method of Dorrer^44^ and Wu^45^, which considers the nonlinearities of the system. Then, Gaussian windowing was performed. The dispersion of the system, as well as the remaining dispersion introduced by the eye, was also corrected using the method introduced in^46^. Finally, the Fourier transform was calculated, resulting in the depth profile of the sample. The reconstructed B-scans and volumes were loaded into ImageJ (Version 1.52p, National Institutes of Health, USA) and motion-corrected using a rigid body transformation of the StackReg plugin (Version July 7, 2011)^47^, followed by an ImageJ 3D median filter with kernel radii of *x*: 0.5 (fast axis), *y*: 0.5 (depth), and *z*: 2 (slow axis).^28^

For the complex-based OCTA calculation, an adapted version of Salas et al.^48^ was used. Due to the long acquisition time, bulk and eye motion artifacts were introduced into the recorded volume. To account for these, both axial motion and transverse motion were corrected. The five consecutive B-scans within a set of repetitions were aligned in the *x*–*y* axis (fast axis—depth) direction with respect to the first B-scan in a set to account for small motion between the five repetitions. To compute the complex-based OCTA image, the phase shift along the slow (scanning) axis, introduced by bulk motion, had to be compensated. For this, the phase difference between consecutive B-scans was calculated by multiplying the complex B-scan with the conjugate of the consecutive one. Then, for the resulting B-scan, the argument of the sum of the complex values along the A-scan direction (depth direction) was computed, generating a vector with an average phase shift value, corresponding to the axial bulk motion, for each fast (scanning) axis position. A moving average filter with a five-pixel window was applied to the vector to reduce phase noise. The average phase shift for each fast axis position was then added to each A-scan of the consecutive B-scans of the set. Low amplitude values (threshold selected empirically) were omitted by setting them to NaN. Finally, pairwise differences among the five bulk-motion-corrected complex B-scans at each slow axis position were computed, resulting in four differential complex B-scans. The average of the absolute values of the four B-scans was computed as one angiographic B-scan and calculated for every set of consecutive B-scans recorded at each slow axis position to retrieve an angiographic volume. To correct for remaining axial shifts along the slow axis direction, a global bulk motion correction was achieved by aligning all the OCTA B-scans axially to the central one of the volume. The transverse distortions introduced by eye motion, such as smooth pursuit, vergence shifts and saccade, were partially corrected by sequentially aligning the B-scans in the fast axis direction.

Supplementary information accompanies the paper on the *Light: Science & Applications* website (http://www.nature.com/lsa).

## Supplementary information

Supplementary Information-Spectral range of AWGs, Intra wafer variations, and compensation strategies
