# Supplementary material for: Toward optical coherence tomography on a chip: in vivo three-dimensional human retinal imaging using photonic integrated circuit-based arrayed waveguide gratings
Source: Light Sci Appl. 2021 Jan 5;10:6. doi: 10.1038/s41377-020-00450-0 (PMC7785745; doi:10.1038/s41377-020-00450-0)
Supplement: Supplementary file 1 — Supplementary Information-Spectral range of AWGs, Intra wafer variations, and compensation strategies [file 41377_2020_450_MOESM1_ESM.docx]

Supplementary information

**Towards optical coherence tomography on a chip: in vivo three-dimensional human retinal imaging using photonic integrated circuit-based arrayed waveguide gratings**

Elisabet A. Rank^1*^, Ryan Sentosa^1^, Danielle J. Harper^1^, Matthias Salas^1^, Anna Gaugutz^1^, Dana Seyringer^2^, Stefan Nevlacsil^3^, Alejandro Maese-Novo^3^, Moritz Eggeling^3^, Paul Muellner^3^, Rainer Hainberger^3^, Martin Sagmeister^4^, Jochen Kraft^4^, Rainer A. Leitgeb^1^, and Wolfgang Drexler^1^

^1^ Center for Medical Physics and Biomedical Engineering, Medical University of Vienna, Waehringer Guertel 18-20/4L, 1090 Vienna, Austria

^2^ Research Centre for Microtechnology, Vorarlberg University of Applied Sciences, Hochschulstrasse 1, 6850 Dornbirn, Austria

^3^ AIT Austrian Institute of Technology GmbH, Gieffinggasse 4. 1210 Vienna, Austria

^4^ ams AG, Tobelbader Strasse 30, 8141 Premstaetten, Austria

^*^ +43 1 40 400 -39 220* Elisabet.rank@meduniwien.ac.at

**Spectral range of AWGs and intra wafer variations**

- **Intra wafer variations**


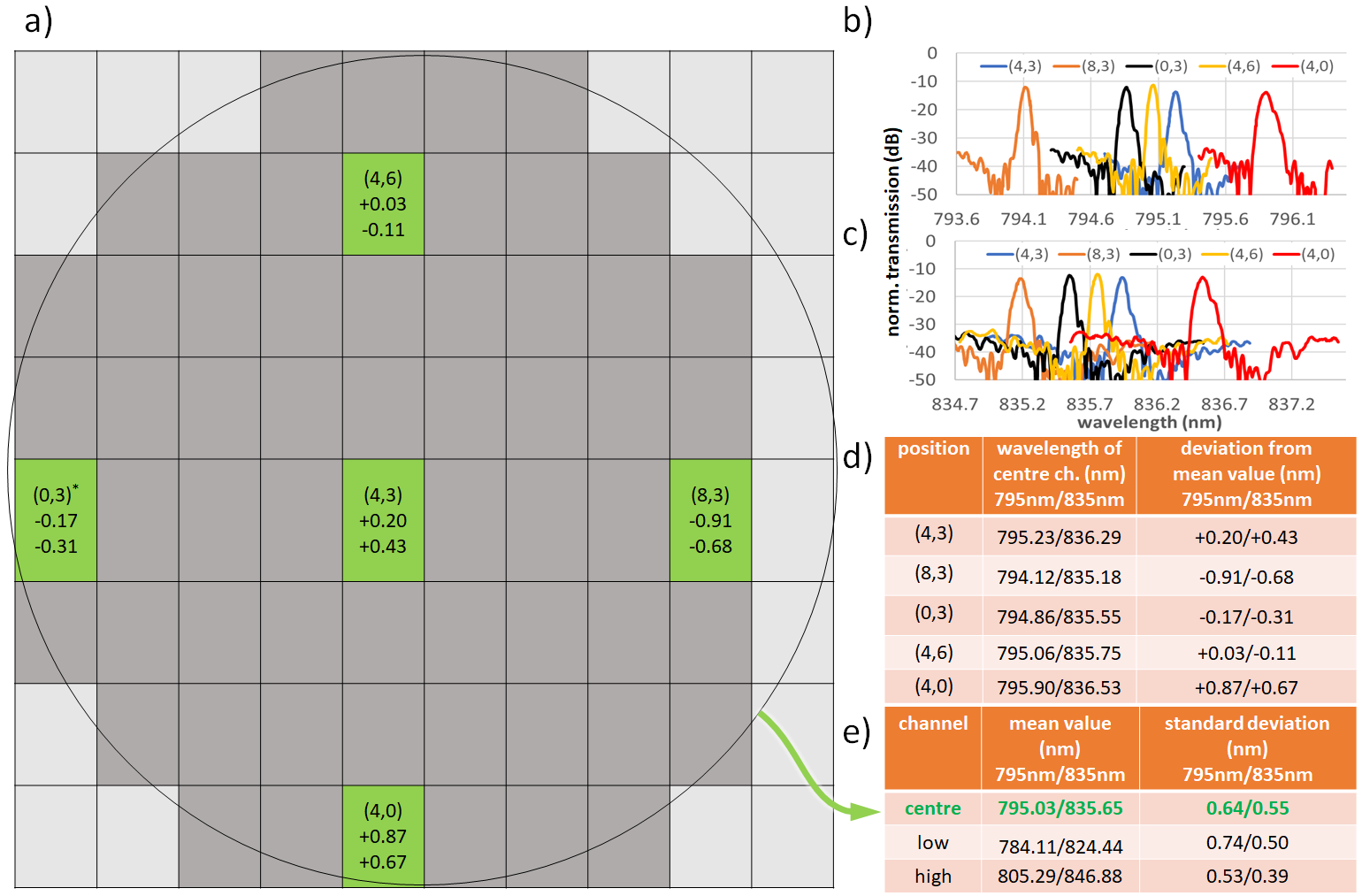


**Figure S1** **Intra-wafer variation of the AWG characteristics**. **a** schematic of the wafer is shown. Five samples at five positions (highlighted in green) were measured. The numbers in the brackets are the x and y coordinates on the wafer (x,y) starting with (0,0). The two other numbers are the difference between the central channel wavelength and the mean wavelength for the spectral ranges of 795nm (central number) and 835 nm (bottom number). The AWG annotated with * is the one used for the measurements in **Fig. S2**. All five center peaks are plotted in **b** for the spectral range centered at 795 nm and **c** for the spectral range centered at 835 nm. **d** summarizes the measured peak wavelengths at the central channel and the deviation from the mean value is provided. **e** summarizes the mean value and standard deviation of all five channels (center, lowest, highest).

**Figure S1** shows the intra-wafer variation of the AWG characteristics. At five positions, as depicted in green in **Fig. S1a**, spanning the entire wafer in the two cardinal directions, the central and outermost channels were measured for two of the spectral ranges: **Fig. S1b** shows all five central channels for the spectral range around 795 nm, **Fig. S1b** shows the same central channels for the spectral range around 835 nm. **Fig. S1c** summarizes the measured central wavelengths of the investigated AWGs and **Fig. S1d** summarizes the mean wavelength and the standard deviation of these as well as the lowest and highest channel, for both spectral ranges. A variation of the spectral position of around $\pm$0.9nm was determined for the AWG 1 design. This variation is similar for both presented AWG designs and will not prevent a future commercialization.

- **Spectral ranges of arrayed waveguide gratings**

As pointed out in the main body of the paper, for the AWG order closest to the design wavelength, the shift is ~16 nm. The measurement of **Fig. S2** explains the characteristic of such an AWG spectrometer in more detail.

**Figure S2 Measurement of the center channel of AWG 1 at the wafer position annotated with * in Fig. S1.**The center channel has a first maximum within this wavelength range at ~794 nm, the next at ~834 nm and at ~881 nm. This means, that the AWG spectrum repeats itself every ~40 - 46 nm. Moreover, from this follows, that the difference of the two AWG types is not 70 nm but 16 nm and the spectral order closest to 850 nm occurs at 834 nm, which means that we observed a shift of the aforementioned 16 nm between designed and fabricated AWG. Since the reoccurring spectra are similar in performance it was an acceptable compromise to use the AWG order at 794 nm which fits well to SLD 1.

A similar shift was observed for the same design on multiple wafers indicating a constant offset between the simulation and the fabrication, rather than fabrication inter-wafer variations. Therefore, the parameters in the simulation model can be adjusted to match the AWG measurements resulting in an improved design more attuned to the fabrication in future runs.

- **Optimization and compensation of wavelength shifts**

An according optimization was performed by the authors for a silica-based, eight channel AWG. For this purpose, PHASAR was incorporated ^1^, where the design wavelength was changed by three nanometers from 1550 nm to 1553 nm to center the spectrum of the fabricated AWG at 1550 nm. The measurements of the fabricated AWGs in **Fig. S3** show, that this correction measure was successful. **Figure S3a** show measurements of the AWG with a designed wavelength of 1500 nm and a measured wavelength of ~1547 nm. **Figure S3b** shows the corrected cpectrum with a design wavelength of 1553 nm and a measured central wavelength of ~1550 nm. The same procedure can be applied to the SiN based AWG, because the optical properties of the waveguide is in PHASAR defined by its effective index, which can be calculated with Eigenmode solvers identical to the silica platform. Nevertheless, the size of the here presented SiN AWG differs largely from the silica AWG, in particular due to the 32 times higher channel count.
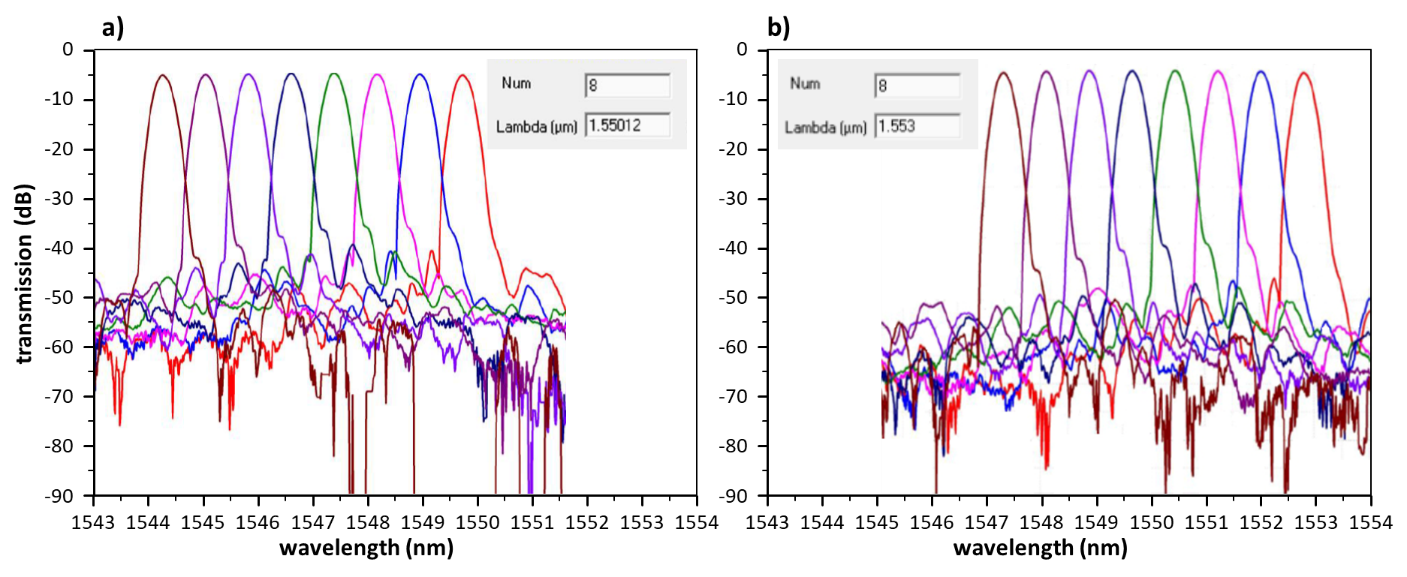


**Figure S3:** **Transmission measurements of an eight channel AWG** with silica as waveguide material for a desired central wavelength in the simulation tool PHASAR of **a** 1550 nm and **b** 1553 nm to compensate the 3 nm shift between the design and measurement.

Therefore, we performed according calculations with PHASAR exemplarily for AWG 1. The results are shown in **Figure S4**. In order to compensate the shift of 16 nm between the current design for 850 nm (**Fig. S4a**), where we measured 834 nm, the central wavelength must be shifted to 866 nm in the design (**Fig. S4b**). The values in the blue boxes are the predefined parameters. The values in the red boxes are the derived AWG parameters, calculated by PHASAR. For the derived parameters, three significant changes are apparent. The number of the phased array waveguides (Na), the length of the input and output coupler (Lf), and the path length difference of the waveguides in the phased array (dL). From the fabrication point of view, the change in the Na has no impact. The change of Lf from 5102.63 *µ*m to 5080.25 *µ*m, as well as the decrease of the dL from 11.55 *µ*m to 11.40 *µ*m are well controllable with state-of-the-art technology. Moreover, we compared the two designs, because an important conclusion of **Fig. S1** is, that the 6 nm shift of the wavelength region of AWG 1 compared to AWG 2 is not a direct consequence of the deviations caused by the fabrication. The reason for the shift is rather a varying impact of the fabrication variations depending on the two different designs. To enable a doubled bandwidth, a completely different phase array design (lengths and distances of the phased array waveguides) had to be employed which influences the overall wavelength shift of a design. Hence, different designs can induce different amount of spectral shift but should be stable for each individual design.

Nevertheless, the comparison of the two layouts revealed, that their footprint, in terms of size, area and profile, are similar (see green dashes in **Fig. S4c** and **Fig. S4d**). From this we follow, that the fabrication of the adapted design will lead to the same deviations as for the current design. Therefore, no unwanted changes of the AWG characteristics will occur, and we expect only the desired shift of 16 nm of the entire spectrum.


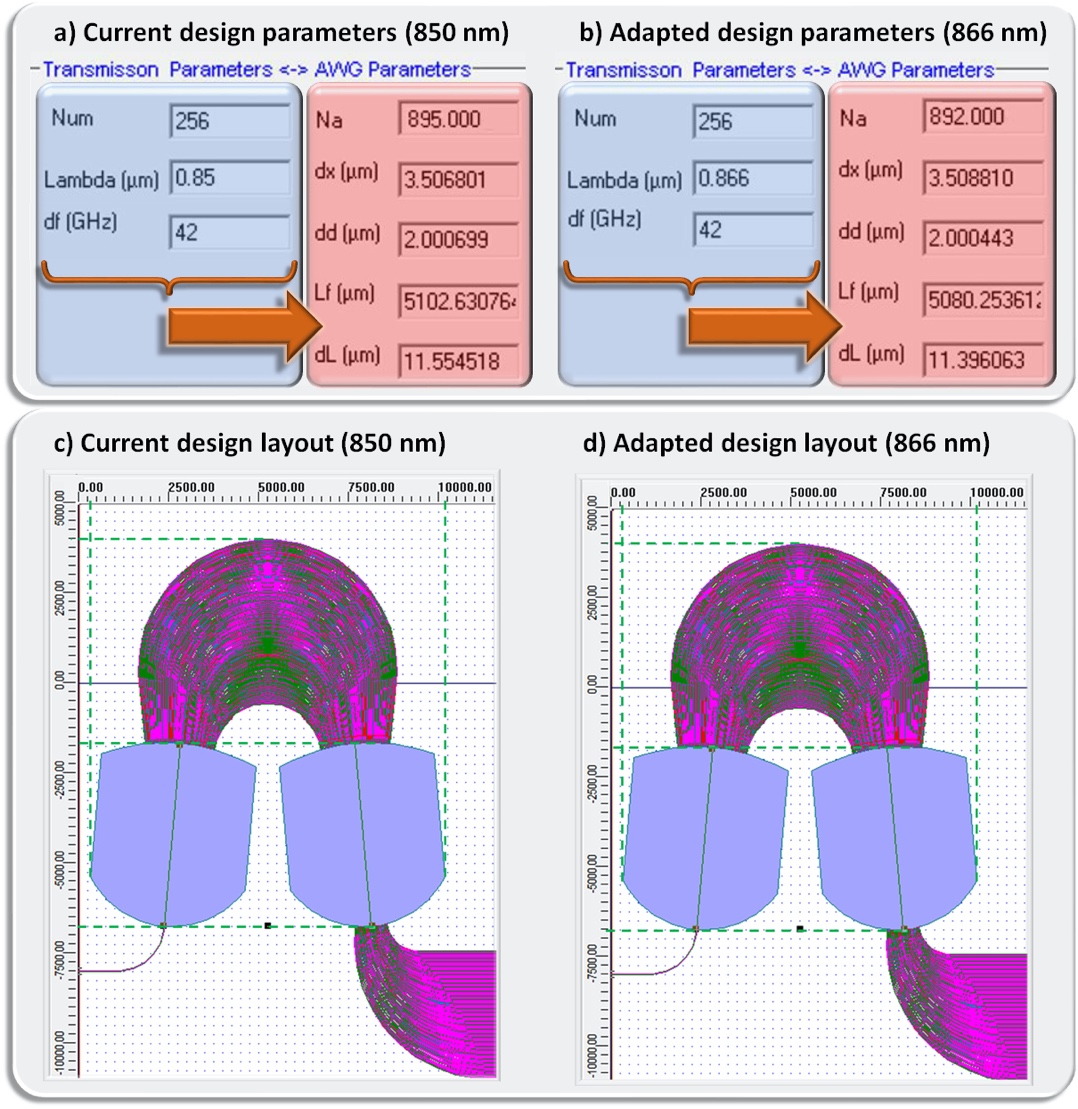


**Figure S4 Comparison of the AWG parameters** for the current, in this paper presented AWG 1 design, and an adapted design at 866 nm design central wavelength, where the measured shift of 16 nm is compensated. **a** shows the AWG parameters for the current AWG 1 design at center wavelength of 850 nm. In **b** the adapted design parameters for 866 nm are depicted. In the blue boxes, the important simulation parameters are given. In the red boxes, the PHASAR simulation results are shown. Num=number of channels; Lambda=wavelength; df=channel spacing provided in GHz; Na=central wavelength; dx=spacing of output waveguides; dd=spacing of waveguides in the phased array; Lf=Length of input and output coupler; dL= path length difference between the waveguides in the phased array. In **c** the according layout for 850 nm design is depicted and in **d** the layout for the adapted 866 nm design. The differences between the two layouts are negligible in terms of overall geometry and footprint (see green dashed lines). This indicates, that the only significant change of the characteristic (of the fabricated adapted design) will be the desired shift of ~16 nm of the entire spectrum.

To facilitate the post fabrication optimization of the AWG design in the simulation, additional measurements were carried out to find likely causes of performance variation. Scanning electron microscopy measurements revealed that the variations were in the typical range for such an i-line stepper-based lithography. However, the variation of the width for specific positions on the wafer did not correlate to the AWG performance variation. While the width variation is radial symmetric, the variation of the AWG characteristic is not (see **Figure S1**). Therefore, in a next step, refractive index measurements of the silica and silicon nitride layers were carried out. These measurements revealed a non-radial difference between maximum and minimum of ~0.0015 for both involved materials: silica and silicon nitride.

First qualitative calculations showed, that such a variation can be responsible for the observed spectral shift of the AWGs. Hence, further optimization of the deposition of the silica and silicon nitride is required in the case that a wavelength precision below $\pm$1 nm is pursued. For example, for silica, several deposition methods are available at the fabrication partner ams AG (ams AG, Tobelbader Strasse 30, 8141 Premstaetten, Austria. Represented by the co-authors M. Sagmeister and J. Kraft). This includes plasma enhanced chemical vapor deposition and high-density plasma-deposition of the SiO_2_, on different tools. SiO_2_ deposition is a commonly used process in CMOS technologies.^2^

Finally, we would like to mention, that a variation or shift of the entire spectrum of up to $\pm$ 7.5 nm can be tolerated and will not hinder an upcoming commercialization. For the final OCT-system an optimized light source can be chosen with respect to the (expected) central wavelength and the bandwidth. In more detail this means, that the difference between the highest wavelength of one spectral AWG order and the lowest wavelength of the next spectral AWG order is 15 nm. This means, that a light source with a bandwidth of $\pm$ 7.5 nm additional spectral range can be employed without an overlap between the light source spectrum and a perturbing, adjacent AWG order. Vice versa, with a stable light source with a 7.5 nm larger bandwidth compared to the AWG (bandwidth ~23 nm), the spectrum of the AWG can vary by $\pm$7.5 nm without any significant degradation with respect to the OCT performance, as long as the other AWG characteristics (number of channels, channel spacing, losses, crosstalk) stay the same.

**References**

1. Smit, M. K. New focusing and dispersive planar component based on an optical phased array. *Electron. Lett.* **24**, 385–386 (1988).

2. ams AG. *CMOS Processes* https://ams.com/process-technology#cmos (2020).
